# Physical activity and functional disability among older adults in Ghana: The moderating role of multi-morbidity

**DOI:** 10.1371/journal.pgph.0001014

**Published:** 2023-03-08

**Authors:** Kofi Awuviry-Newton, Mary Amponsah, Dinah Amoah, Pablo Villalobos Dintrans, Adjeiwa Akosua Afram, Julie Byles, Jacob Rugare Mugumbate, Paul Kowal, Nestor Asiamah

**Affiliations:** 1 African Health and Ageing Research Centre (AHaARC), Winneba, Ghana; 2 College of Health and Biomedicine, Victoria University, Victoria, Australia; 3 Centre for African Research, Engagement and Partnerships (CARE-P), The University of Newcastle, Newcastle, Australia; 4 School of Health Sciences, University of Tasmania, Hobart, Australia; 5 Programa Centro Salud Pública, Facultad de Ciencias Médicas, Universidad de Santiago, Santiago, Chile; 6 Millennium Institute for Caregiving Research (MICARE), Chile; 7 Centre for Women’s Health Research, Hunter Medical Research Institutes, The University of Newcastle, Newcastle, Australia; 8 School of Health and Society, University of Wollongong, Wollongong, Australia; 9 International Health Transitions, Canberra, Australia; 10 Division of Interdisciplinary Research and Practice, School of Health and Social Care, University of Essex, Essex, United Kingdom; African Population and Health Research Center, KENYA

## Abstract

Knowledge about how physical activity levels relate to functional disability is essential for health promotion and planning older adults’ care or rehabilitation. The risk of living with one or more chronic health conditions increases with increasing age in lower and higher income countries–many of which are associated with physical inactivity. We conducted a cross-sectional study to examine the moderating role of multimorbidity on physical activity and its measures on functional disability among older adults in Ghana. Data from WHO’s Study on global AGEing and adult health Ghana Wave 2 with a sample of 4,446 people aged 50+ years was used for this study. Functional disability was assessed using the 12-item WHO Disability Assessment Schedule 2.0. Three categories of physical activity levels were used: vigorous intensity, moderate intensity, and walking. Past month diagnosis by a doctor was used to assess the presence of a chronic condition, and the presence of two or more conditions was used to define multi-morbidity. Logistic regressions with a post hoc interactional tests were used to examine the associations. Overall, physical activity had a significant association with functional disability (OR = 0.25, 95%CI; 0.12, 0.32). A similar relationship was found for vigorous-intensity (OR = 0.19, 95%CI: 0.12, 0.29), moderate-intensity (OR = 0.19, 95%CI: 0.15, 0.25) and walking (OR = 0.41, 95%CI: 0.33, 0.51). Older adults living with one condition and physically active were 47% less likely to experience functional disability compared with the less active counterparts living with at least two chronic conditions. Among the three measures of physical activity, multimorbidity moderated the relationship between walking and functional disability. Future strategies for meeting the health and long-term care needs of older adults, particularly those living with only one chronic condition in Ghana should consider encouraging walking. Policies, financial assistance, family, and community level interventions aimed to promote and sustain physical activity among older adults should be a priority for stakeholders in Ghana.

## Introduction

The call for implementing long-term care systems for older adults in every country, including Ghana [1], requires an understanding of the relationship, among others, between physical activity and functional disability. Physical activity has proven to be essential in maintaining health and addressing older adults’ health and long-term care needs in western countries [2, 3]. Physical activity is defined as any bodily movement produced by skeletal muscles that require energy expenditure either through moderate or vigorous-intensity activities or walking [4]. Related to this, functional disability refers to the difficulty in completing activities relating to cognition, mobility, self-care, getting along with others, life activities and participation in society [5–7].

Although evidence on the effects of physical activity on functional disability in African countries such as Ghana is limited, there is more information from high-income countries. These studies have demonstrated how physical activity leads to a reduction in functional disability [8–11] by reducing the occurrence of chronic diseases [12]. For example, Kim, Park [13] revealed that engaging in a form of physical activity can lead to decrease in the incidence of depression later in life for adults in South Korea. Also, regular physical activity improves the gait and balance of older adults, consequently reducing the incidence of falls [14] as well as improving motor and auditory attention [15]. A meta-analysis by Tak et al. [16] revealed that an increase in physical activity led to delayed progression of functional disability in older adults; however, they did not find any difference in the rate of decline in functional disability following an increase in physical activity by either an older adult with or without disability [16]. Finally, evidence from 47 low-and middle-income countries reports that high physical activity was associated with less severe subjective memory and learning difficulties [17].

In both lower and higher income countries, multiple factors are known to moderate the relationship between physical activity and functional disability among older adults. Chronic pain is a key factor responsible for physical inactivity [18, 19], and a risk factor for functional disability in older adults [20]. Other known moderating factors include poor health status [21, 22], residing in urban areas [21, 23–25], being an older female [26, 27], educational level [22, 28], and marital status [18, 29, 30].

In sub-Saharan African countries, evidence on how these moderators influence the relationship between physical activity measures and functional disability is scant, despite its relevance in determining healthy ageing. Available evidence from a study conducted in low-and middle-income countries suggests that multi-morbidity may moderate the association between physical activity and functional disability [17]. This study aims to evaluate the interactional role of multi-morbidity on the association between physical activity and functional disability, controlling for a number of confounding variables. The novelty of the present study lies in its intention to provide baseline evidence on the relationship between physical activity, functional disability and multimorbidity among older adults in Ghana.

## Methods

### Ethics statement

Ethical approval for this study was obtained from the WHO Ethical Research Committee (#ID3925). Consent for participants were obtained before the commencement of the study [31].

### Study sample

We used data from the Study on global AGEing and adult health (SAGE) Ghana Wave 2 conducted between 2014/2015. SAGE is a Multi-Country (Ghana, South Africa, China, India, Mexico, and Russia) longitudinal study that employed multistage cluster sampling strategies. The University of Ghana Medical School through the Department of Community Health, and in collaboration with the World Health Organization (WHO), implemented the SAGE Wave 2 in Ghana. The current study used a sample size of 4,446 participants (+50 years) who answered all 12 questions on functional disability. Details about the methodology and other relevant information on the study are available elsewhere [32].

### Variables

#### Functional disability

Functional disability was defined using the 12-item version of the WHO Disability Assessment Schedule (WHODAS 2.0), which classifies responses into five disability categories: none, mild, moderate, severe, and extremely severe. In its full version, the WHODAS 2.0 contains 12 questions from six domains: cognition, mobility, self-care, getting along, life activities, and participation in society [33]. S1 Appendix contains the questions included in the analysis. WHODAS 2.0 was scored on a scale of 0 to 100, with a lower score implying point for determining the severity of the disability [11, 34, 35]. Participants scoring <90.18% were denoted as “no disability” and participants who score > = 90.18% were denoted as “with a disability.”

#### Physical activity

Physical activity was measured with three separate items including vigorous activity, moderate activity, and walking. Vigorous-intensity activity was measured by the question *“Does your work involve vigorous-intensity activity that causes large increases in breathing or heart rate*, *[like heavy lifting*, *digging*, *or chopping wood] for at least 10 minutes continuously (Yes/No)*? The question *“Does your work involve moderate-intensity activity that causes small increases in breathing or heart rate [such as brisk walking*, *carrying light loads*, *cleaning*, *cooking*, *or washing clothes] for at least 10 minutes continuously*?*” (Yes/No)* was used to measure work-related moderate-intensity activity. We used the question *“Do you walk or use a bicycle (pedal cycle) for at least 10 minutes continuously to get to and from places*?*” (Yes/ No)* to measure older adults’ engagement in walking.

Additionally, these three measures were scored and aggregated into two response categories; *yes* (engages in at least one of the three measures of physical activity) and *no* (engages in none of the three measures). The Cronbach’s α of the three physical activity items combined was 0.61.

#### Multimorbidity

The question *“Have you ever been diagnosed with/told you have … in the past month*? *(Yes/No)* was used to identify the presence of each of the 11 chronic conditions including stroke, hypertension, injuries, depression, diabetes, angina, arthritis, chronic lung disease, asthma, cataract, and oral health among older adults. Responses were combined and a variable capturing the presence of different conditions was generated to measure multimorbidity (1 = *no condition*, *2 = one chronic condition and 3 = at least two conditions*).

#### Covariates

Sociodemographic and health confounding variables included in the analysis were age (continuous), sex (1 = male, 2 = female), marital status (1 = never married, 2 = married/cohabiting, 3 = separated/divorced, 4 = widowed), education (1 = less than primary school, 2 = primary education completed, 3 = senior high completed, 4 = university degree/post), location of residence (1 = rural, 2 = urban) and self-reported health status (1 = good, 2 = moderate, 3 = bad). The health status variable was excluded from the model as it was not statistically significant at p>0.05.

### Data analysis

Using STATA version 16, frequency, and percentages, and means and standard deviations were used to describe the variables in the study. Second, bivariate analyses were performed through chi-square, Fisher’s test, and t-test to test relationships between independent variables and dependent variables. Finally, bivariate, and multivariate logistic regression were performed to estimate the crude and adjusted odds ratios (OR) and 95% confidence intervals (CI) for the associations between physical activity and functional disability, and the moderation effect (interaction) of multimorbidity in the relationship.

### ‘Inclusivity in global research’

Additional information regarding the ethical, cultural, and scientific considerations specific to inclusivity in global research is included in the Supporting Information.

## Results

### Descriptive statistics

The results in Table 1 show the univariate and bivariate analysis of independent variables in relationship to functional disability. Most of the participants were females (58.9%). The mean age of participants was 57.6 years but the sample of people with functional disability was older, reaching a mean of 74 years (SD,12.2). The prevalence of functional disability among females was higher than in males (64.5% vs 35.5%). A high proportion of functional disability was reported among widowed older adults (46.5%), higher in rural compared to urban areas. A higher prevalence of functional disability was reported among older adults who completed senior high education (39.5%). Regarding multimorbidity, a high prevalence of functional disability was reported among older adults living with at least two chronic conditions (45.9%, p<0.001). Similarly, older adults with self-reported bad health experienced a high prevalence of functional disability (68.2%, p<0.001).

**Table 1:** Univariate and bivariate analysis of independent variables and functional disability.

Independent variables	Overall	Functional disability		p-value
	N (%)	No disability, N (%)	With disability, N (%)	
**Age** (Mean, SD)	57.6±16.7	55.0±16.0	74.1±12.2	<0.001
**Sex**				<0.01
Male	1,826 (41.1)	1,658 (41.7)	168 (35.5)	
Female	2,620 (58.9)	2,315 (58.3)	305 (64.5)	
**Marital status**				<0.001
Never married	416 (9.36)	409 (10.3)	7 (1.48)	
Married/cohabiting	2,555 (57.5)	2,366 (59.6)	189 (40.0)	
Separated/divorce	499 (11.2)	442 (11.1)	57 (12.1)	
Widowed	976 (21.9)	756 (19.0)	220 (46.5)	
**Location of residence**				0.331
Rural	2,624 (59.0)	2,335 (58.8)	289 (61.1)	
Urban	1822 (41.0)	1,638 (41.2)	184 (38.9)	
**Education**				0.056
Less than primary school	610 (23.6)	559 (23.0)	51 (32.5)	
Primary education completed	664 (25.7)	629 (25.9)	35 (22.3)	
Senior high completed	1,168 (45.2)	1,106 (45.6)	62 (39.5)	
University degree/post	142 (5.50)	133 (5.48)	9 (5.73)	
**Health status**				<0.001*
Good	627 (18.4)	625 (19.8)	2 (0.76)	
Moderate	2,448 (71.7)	2,366 (75.1)	82 (31.1)	
Bad	341 (9.98)	161 (5.11)	180 (68.2)	
**Multimorbidity**				<0.001
No morbidity	2,365 (53.2)	2,193 (55.2)	172 (36.4)	
Only one morbidity	529 (11.9)	445 (11.2)	84 (17.8)	
2 or more morbidities	1,335 (33.6)	1,335 (33.6)	217 (45.9)	
**Physical activity (PA)**				
Vigorous-intensity activity				<0.001
Yes	1,346 (30.5)	1,324 (33.6)	22 (4.69)	
No	3,069 (69.5)	2,622 (66.5)	447 (95.3)	
Moderate-intensity activity				<0.001
Yes	2,576 (58.4)	2,489 (63.1)	87 (18.6)	
No	1,839 (41.7)	1,457 (36.9)	382 (81.5)	
Walk				<0.001
Yes	2,878 (65.2)	2,684 (68.0)	194 (41.4)	
No	1,537 (34.8)	1,262 (32.0)	275 (58.6)	
PA (Overall)				<0.001
Yes	3,392 (76.3)	3,182 (80.1)	210 (44.4)	
No	1,054 (23.7)	791 (19.9)	263 (55.6)	
Functional disability				
No	3,973 (89.4)	-	-	-
Yes	473 (10.6)	-	-	-

*Fisher’s p-value

Older adults who engaged in physical activity of any kind reported lower functional disability compared to those who were not active (44.4% vs 58.6, p<0.001). A similar result was revealed in the engagement of three specific measures of physical activity (vigorous (4.69%), moderate (18.6%) or walking (41.4%) compared to those who do not engage in these activities (p<0.001). The prevalence of functional disability among older adults was approximately 11%.

### Primary results

In this section, we discuss the relationship between overall physical activity and functional disability, while controlling for potential confounding variables including age, sex, marital status and multimorbidity (Table 2). In the unadjusted model (column 1), older adults who were physically active were 80% less likely to experience functional disability (OR = 0.20, 95%CI; 0.16, 0.24). This effect is still observed in the adjusted models (columns 2–6), showing robust evidence of a negative significant relationship between physical activity and functional disability (OR = 0.25, 95%CI; 0.12, 0.32), with minimal changes in the odds ratios. It is important to note that physical activity is measured in its aggregate form, hence the results do not show the potential different effects of different measures of physical activity.

**Table 2:** Relationship between physical activity and functional disability adjusted for confounders.

	Model 1	Model 2	Model 03	Model 4	Model 5	Model 6
**Physical activity** (PA)	0.20 (0.16, 0.24)***	0.26 (0.21, 0.32)***	0.20 (0.16, 0.24)***	0.19 (0.16, 0.24)***	0.21 (0.17, 0.25)***	0.25 (0.12, 0.32)***
Age		1.10 (1.09, 1.11)***				1.10 (1.09, 1.11)***
**Sex**						
Male			1			1
Female			1.24 (1.01, 1.52)*			1.35 (1.03, 1.76)*
**Marital status**						
Never married				0.18 (0.08, 0.38)***		0.41 (0.18, 0.93)*
Married/cohabiting				1		1
Separated/divorce				1.78 (1.28, 2.46)***		1.54 (1.08, 2.21)*
Widowed				3.48 (2.79, 4.34)***		1.37 (1.03, 1.83)*
**Multimorbidity**						
No morbidity					1	1
Any one morbidity					2.13 (1.59, 2.85)***	1.02 (0.91, 1.41)
2 or more morbidities					1.92 (1.54, 2.39)***	1.39 (1.09, 1.77)***

Notes: Health status was removed leaving multi-morbidity because the final model was better without it. Model 1- PA and functional disability; Model 2 –PA, functional disability and age; Model 3- PA, functional disability and sex; Model 4—PA, functional disability and marital status; model 5—PA, functional disability and multimorbidity; model 6—PA, functional disability, age, sex, marital status and multi-morbidity.

***, **, * denote significant levels at 1%, 5% and 10%, respectively.

### Interactions of multimorbidity on the association between physical activity and functional disability

As part of the objective of this study, we estimated the interactions of the impact of morbidity on the relationship between physical activity and functional disability. The results revealed that having at least one chronic condition was associated with more disability; while the interaction between physical activity and multi-morbidity showed no significant effect on functional disability, except for people who experienced one morbidity: for this group, older people who engaged in overall physical activity were 47% less likely to experience functional disability (OR = 0.53, 95%CI; 0.29, 0.96) compared to their counterparts (Tables 3 and 4).

**Table 3:** Effects of confounding on the relationship between physical activity and functional disability.

	[1]	[2]	[3]	[4]	[5]	[6]	[7]	[8]	[9]	[10]	[11]	[12]	[13]	[14]	[15]	[16]	[17]	[18]
Vigorous	0.10 (0.06, 0.15)***	0.17 (0.11, 0.27)***	0.10 (0.06, 0.15)***	0.11 (0.07, 0.18)***	0.10 (0.07, 0.16)***	0.19 (0.12, 0.29)***												
Moderate							0.13 (0.11, 0.17)***	0.21 (0.16, 0.27)***	0.13 (0.10, 0.16)***	0.13 (0.10, 0.17)***	0.14 (0.11, 0.18)***	0.19 (0.15, 0.25)***						
Walk													0.33 (0.27, 0.40)***	0.41 (0.33, 0.50)***	0.34 (0.28, 0.41)***	0.33 (0.27, 0.41)***	0.34 (0.28, 0.41)***	0.41 (0.33, 0.51)***

Note:[1]–Model 1; [2]–Model 2; [3]–Model 3; [4]–Model 4; [5]–Model 5; [6]–Model 6; [7]–Model 7; [8]- Model; [9]–Model 9; [10]–Model 10;: [11]–Model 11; [12]–Model1 2; [13]–Model 13; [14]–Model1 4; [15]–Model 15; [16]–Model6 6; [17]–Model 17; [18]- Mode8. Model 1- Vigorous and functional disability; Model 2 –Vigorous, functional disability and age; Model 3- Vigorous, functional disability and sex; Model 4—Vigorous, functional disability and marital status; model 5—Vigorous, functional disability and multimorbidity; model 6—Vigorous, functional disability, age, sex, marital status and multi-morbidity; Model 7- Moderate and functional disability; Model 8 –Moderate and functional disability and age; Model 9- Moderate and functional disability and sex; Model 10—Moderate and functional disability and marital status; model 11—Moderate and functional disability and multimorbidity; model 12—Moderate and functional disability, age, sex, marital status and multi-morbidity; Model 13- Walk and functional disability; Model 14 –Walk and functional disability and age; Model 15- Walk and functional disability and sex; Model 16—Walk and functional disability and marital status; model 17—Walk and functional disability and multimorbidity; model 18—Walk and functional disability, age, sex, marital status and multi-morbidity.

***, **, * denote significant levels at 1%, 5% and 10%.

**Table 4:** Multimorbidity moderation on physical activity association with functional disability.

	[1]	[2]	[3]	[4]	[5]
PA (Overall)	0.20 (0.16, 0.24)***				
Vigorous	0.10 (0.06, 0.15)***				
Moderate	0.13 (0.11, 0.17)***				
Walking	0.33 (0.27, 0.40)***				
**Multimorbidity**					
No morbidity					
Any one morbidity					
2 or more morbidities					
PA*Multi-morbidity					
No morbidity		1			
PA*Any one morbidity		0.53 (0.29, 0.96)*			
PA*2 or more morbidities		0.76 (0.49, 1.17)			
Vigorous*Multimorbidity					
No morbidity			1		
Vigorous*Any one morbidity			0.83 (0.22, 3.04)		
Vigorous*2 or more morbidities			0.48 (0.18, 1.29)		
Moderate*Multimorbidity					
No morbidity				1	
Moderate*Only one morbidity				0.68 (0.32, 1.45)	
Moderate*2 or more morbidities				0.79 (0.47, 1.33)	
Walk*Multimorbidity					
No morbidity					1
Walk*Only one morbidity					0.42 (0.23, 0.76)*
Walk*2 or more morbidities					0.66 (0.43, 1.02)

Model 1 –Odds ratio between the variables and functional disability; Model 2 –interaction between overall PA and multi-morbidity; Model 3—interaction between overall vigorous and multi-morbidity; Model 4—interaction between moderate-intensity activity and multi-morbidity; Model 5—interaction between walk and multi-morbidity

### Sensitivity analysis

This analysis was conducted to determine which type of work-related physical activity (vigorous-intensity activity, moderate-intensity activity, and walking) is more likely to affect functional disability. Table 3 reports the association of these activities on functional disability while Table 4 reports the interaction between multimorbidity and physical activity association on functional disability.

Work-related vigorously intense activity was significantly associated with functional disability (Table 3, columns 1–6). Older adults who engaged in vigorously intense activity were 90% less likely to experience functional disability (OR = 0.10, 95%CI: 0.06, 0.15) compared to their counterparts. When adjusted for all confounding included in the study (Table 3, column 6), vigorously intense activity significantly and independently was associated with functional disability (OR = 0.19, 95%CI: 0.12, 0.29).

For moderate intensity (Table 3, columns 7–12), there was a significant association with functional disability (OR = 0.13, 95%CI: 0.11, 0.17). After controlling for confounding variables, the significant association remained (OR = 0.19, 95%CI: 0.15, 0.25).

Similarly, older adults who engaged in walking were 67% less likely to experience functional disability (OR = 0.33, 95%CI: 0.27, 0.40) compared to their counterparts (unadjusted column 13 in Table 3). A similar association was found when controlling for all confounders in Model 18 in Table 3 (OR = 0.41, 95%CI: 0.33, 0.51).

Table 4 presents the results of including multimorbidity as a moderator in the relationship between functional disability and physical activity. While no evidence existed that multimorbidity moderated the relationships between functional disability associations with vigorous- moderate activity, we found significant evidence of the impact of multimorbidity on the association between walking and functional disability. That is, older adults who experience only one chronic condition and engaged in walking were 58% less likely to experience functional disability (OR = 0.42, 95%CI: 0.23, 0.76) compared to their counterparts. This is evidence that engaging in a physical activity, particularly walking, when having any one chronic condition, decreases the likelihood of experiencing functional disability.

## Discussion

The current study revealed that older adults with physical activity engagement were 75% less likely to experience functional disability. Physical activity measures such as vigorous-intensity, moderate-intensity and walking were independently significantly associated with functional disability among older adults. Older adults living with only one chronic condition and engaging in walking were 58% less likely to experience functional disability compared to those with no or at least two chronic conditions. The current study adds to the available literature that multimorbidity moderates the relationship between physical activity (walking) and functional disability. This finding extends the existing evidence that significant number of older adults in low- and middle-income countries live with functional disability [11], by adding that engaging in specific physical activity such as vigorous, moderate or walking can contribute to the improvement of the functional ability of older adults. The result is consistent with previous studies from developed countries on how engagement in physical activity reduces the incidence of chronic diseases and depression later in life [12, 13], ultimately reducing premature mortalities [36–38], and improving quality of life [39].

The current study revealed a negative relationship between physical activity and functional disability. The examination of the intensity of physical activity that yielded a reduced functional disability revealed that all measures (vigorous, moderate, or walking) resulted in an improvement in older adults ‘functional ability, implying that engagement of any form of physical activity is more likely to lead to reduced functional disability among older adults [13, 15, 20].

Even though evidence has shown that poor health conditions are barrier to physical activity among older adults [21, 22], the current study adds that engagement of physical activity is effective when recommended for older adults with only one chronic condition as they are 47% less likely to experience functional disability as compared to their counterparts. With regards to the intensity of physical activity recommended to achieve a reduction in functional disability, the current study revealed that older adults who experienced only one chronic condition and engaged in walking have better chance of minimizing incidence of functional disability. This finding partly supports a recent study [40] which recommends physical activity for older adults with chronic conditions as it improves functional ability, however, the intensity effective to achieve a reduced functional disability was proposed to be moderate to vigorous physical activity as opposed to light physical activity. The disparity could be due to the nature, duration, and severity of the condition which was not specified. It can therefore be explained that poor health status is not absolutely a barrier to physical activity among older people but the need for sensitization of the benefits of physical activity is paramount [28]. If the benefits of physical activity are made known, older adults may tend to see poor health as a motivation to engage in physical activity than taking medications as reported in a previous study [41]. In this study, the finding that older adults who participated in walking and living with at least two chronic conditions had no association with functional disability is understandable as pains associated with these conditions is usually intense making impact of physical activity such as walking on physical activity insignificant.

Despite the benefits outlined by this study, there are however some limitations. First, data on the type of morbidities were not available as its inclusion would have provided an in-depth understanding of the relationship. Third, the cross-sectional nature of the study limits it in determining the causality of the relationship; future studies using longitudinal research methods can help in understanding how this relationship change over time.

## Conclusions and implications

Physical activity particularly, walk, should be encouraged among older adults, even for those with some health issues, as evidence proves it associates with a decrease in functional disability. This can be achieved through a regular creation of awareness through media, campaigns, commemoration of health events on the need for older adults with one form of morbidity to engage in physical activity. In addition, medical personnel attending to older adults with morbidities should be sensitized on the need to incorporate physical activity into the routine care or post discharge care in a bid to reduce functional dysfunction. Policy interventions should therefore incorporate a built environment (sidewalks) that will facilitate walking, organize regular community activities or programs for elderly adults that involves physical activities. Moreover, caregivers should encourage older adults to take part in community meetings or family functions as this can encourage walking to improve the quality of life of older adult. Policies, public health interventions, and financial assistance aimed to promote and sustain physical activity among older adults should be a priority for stakeholders of rehabilitation of older adults receiving long-term care services of any kind in Ghana. The current finding does not only benefit Ghana, but also other low-and middle-income countries seeking to promote healthy ageing.

## Supporting information

S1 Appendix List of the 12 variables included in the WHODAS 2.0 score. (DOC)

S1 Data Minimal datasets. (XLSX)

S1 Text Inclusivity in global research. (DOCX)
