# Supplementary material for: Physical activity and functional disability among older adults in Ghana: The moderating role of multi-morbidity
Source: PLOS Glob Public Health. 2023 Mar 8;3(3):e0001014. doi: 10.1371/journal.pgph.0001014 (PMC10021534; doi:10.1371/journal.pgph.0001014)
Supplement: S1 Appendix — (DOC) [file pgph.0001014.s001.DOC]

**S1 Appendix Supporting information**

**List of the 12 variables included in the WHODAS score and cut points**

| **In the last 30 days how much difficulty do you have in:** | |
| --- | --- |
| **…learning a new task, for example, learning how to get to a new place?** | None = **0**, Mild = **1**, Moderate = **2**, Severe = **3**, Extreme/cannot = **4** |
| **…making new friendships or maintaining current friendships?** |  |
| **…dealing with strangers?** |  |
| **…standing for long periods, such as 30 min?** |  |
| **…taking care of your household responsibilities?** |  |
| **…joining community activities (for example, festivities, religious or other activities) in the same way as anyone else can?** |  |
| **…concentrating on doing something for 10 min?** |  |
| **…walking long distance, such as one kilometer?** |  |
| **…bathing/washing your whole body?** |  |
| **…getting dressed?** |  |
| **…performing your day to day work?** |  |
| **In the last 30 days, how much have you been emotionally affected by your health condition(s)?** |  |
